# *Belarima violacea* (Lucas, 1847) (Coleoptera, Chrysomelidae), a new genus and species for the European fauna

**DOI:** 10.3897/zookeys.1031.61846

**Published:** 2021-04-15

**Authors:** Michele Violi, Paola D’Alessandro, Maurizio Biondi

**Affiliations:** 1 Via al Forte 8, 42027 Montecchio Emilia (RE), Reggio Emilia, Italy Unaffiliated Reggio Emilia Italy; 2 Department of Health, Life and Environmental Sciences, University of L’Aquila, Via Vetoio snc, 67100, Coppito, L’Aquila, Italy University of L’Aquila L’Aquila Italy

**Keywords:** *Belarima violacea*, Chrysomelidae, Europe, Galerucinae, Galerucini, Italy, North Africa

## Abstract

*Belarima violacea* (Lucas) is an uncommon species of the Galerucini tribe (Coleoptera, Chrysomelidae, Galerucinae) distributed in Algeria, Morocco and Tunisia, and recorded here for the first time for the European fauna. One male and one female were found, not far from each other, wandering on the sand among the vegetation of the shifting dunes of the Tuscan coast (Rosignano Solvay, Spiagge Bianche). Some hypotheses are proposed to explain the presence of *B. violacea* on the Italian coast. Morphological descriptions of external habitus, aedeagus and spematheca, the latter here described for the first time, are also provided, accompanied by micro-photographs.

## Introduction

Galerucinae are a large subfamily of Chrysomelidae, including about 15,000 species comprised in more than 1100 genera, of which more than 500 genera and about 8000 species in the tribe Alticini, and approximately 540 genera and 7200 species in the tribe Galerucini (Nadein and Bezděk 2014; Nie et al. 2017). Galerucini are widespread in all zoogeographic regions, and occur with 13 genera and 123 species in Europe (Beenen 2013, as Galerucinae).

The genus *Belarima* Reitter, 1913, with the species *violacea* (Lucas, 1847), is here recorded for the first time for the European fauna. *Belarima* currently includes two uncommon species: *B. violacea* from Algeria, Morocco and Tunisia, and *B. obliqua* Beenen, 2019, recently described from Algeria. This genus is separated from *Arima* Chapuis, 1875 by the absence of a basal pronotal margin, which in *Arima* is instead finely margined. In addition, *Belarima* shows some costae on the elytra, absent in *Arima*. Beenen (2019) instead considers *Belarima* as more related to *Galeruca* Geoffroy, 1762, because *Arima* has the sides of the abdominal tergites swollen while they are simple in *Belarima*, as in *Galeruca*. However, *Belarima* lacks the apical spurs on the tibiae, whereas they are present in *Galeruca* (Beenen 2019).

## Methods

The specimens were examined, measured and dissected using a Leica M205C stereomicroscope. Photographs were taken using a Leica DFC500 camera and composed using Zerene Stacker version 1.04. Scanning electron micrographs were taken using a Hitachi TM-1000. Terminology follows D’Alessandro et al. (2016) for the median lobe of the aedeagus, and Bezděk (2015) and Rodrigues and Mermudes (2015) for the spermatheca. Geographical coordinates for the localities are reported in degrees, minutes and seconds (WGS84 format).

### Abbreviations for biometry

**LA** numerical sequence proportional to length of each antennomere;

**LAED** length of aedeagus;

**LAN** length of antennae;

**LB** total length of body (from apical margin of head to apex of abdomen);

**LE** length of elytra;

**LP** medial length of pronotum;

**LSP** maximum length of spermatheca;

**WE** maximum combined width of elytra;

**WP** maximum width of pronotum.

## Results

### Belarima violacea

Taxon classificationAnimaliaColeopteraChrysomelidae

(Lucas)

9BD1DB54-5916-51FE-BDBC-D5C5C3D6A8D1

Adimonia violacea Lucas, 1847: plate 44, fig. 7a–c; Lucas 1849: 540–541; Joannis, 1865: 9, 18.
Belarima violacea (Lucas): Warchalowski 2010: 634, pl. LXXV, photo 669; Beenen 2019: 2–4, figs 2, 3b; Beenen 2010: 445.
Galeruca violacea (Lucas): Jolivet, 1967: 330 (biology).

#### New material examined.

Italy, Tuscany (Livorno), Rosignano Solvay, Spiagge Bianche, 43°22'27.58"N, 10°26'21.27"E, 22.iii.2019, M. Violi leg., 1♂ and 1♀ (University of L’Aquila).

#### Collecting locality.

**Figure 1.**
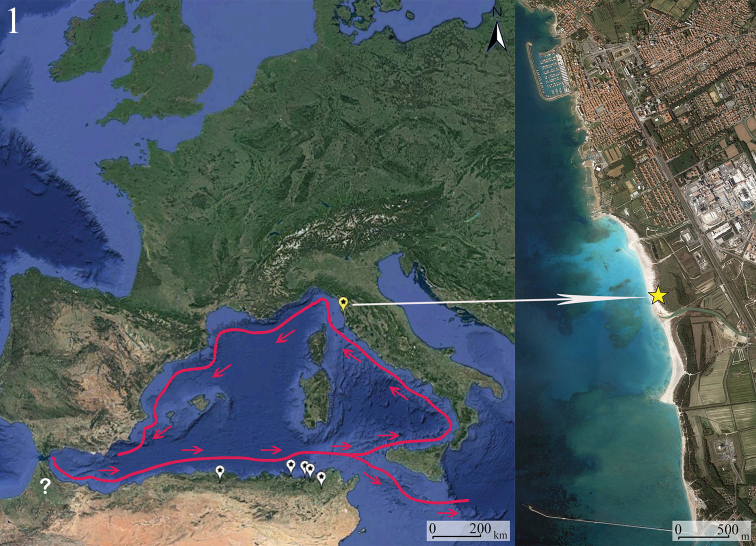
Distribution map of *Belarima violacea* (Lucas). Red line: Algerian current (see text).

One male and one female of *B. violacea* were found, not far from each other, wandering on the sand among the vegetation of the shifting dunes of the Spiagge Bianche (Ligurian Sea, Tuscan coast) (Fig. 1). This site is probably the best preserved of the entire beach, away from the aphytoic belt, that is the vegetation-free zone closest to the water, disturbed in summer by bathers and periodic cleaning. The vegetation consists exclusively of herbaceous essences, mainly *Ammophila arenaria arundinacea* H. Lindb. (Poaceae). On the shoreline, and near the place of the finding, there were numerous trunks, branches and other plant debris carried by the storms. The area is part of the Mediterranean macrobioclimate, low meso-Mediterranean belt and low sub-humid umbrotype (Bertacchi et al. 2016). The finding of the specimens occurred around 5.00 pm on a sunny day with sparse clouds. The site was in the portion of the dunes between the mouths of the Fine and Fosso Bianco rivers. The characteristic white color of the sand is due mainly to the waste deposits derived from the production of calcium carbonate and calcium bicarbonate by the Solvay chemical industrial center (opened in 1916 and still in operation), which is located about a hundred meters behind the place where *B. violacea* was found. About 1.6 km north lies the village of Rosignano Solvay with the tourist port of Cala de’ Medici; about 2.4 km south is the commercial harbor of Vada, a docking point for LNG and ethylene tankers whose contents are destined for Solvay. These two sites would therefore constitute the closest sources for a possible anthropic introduction of the species to this area.

#### Description of the specimens and differential diagnosis.

**Figures 2–4.**
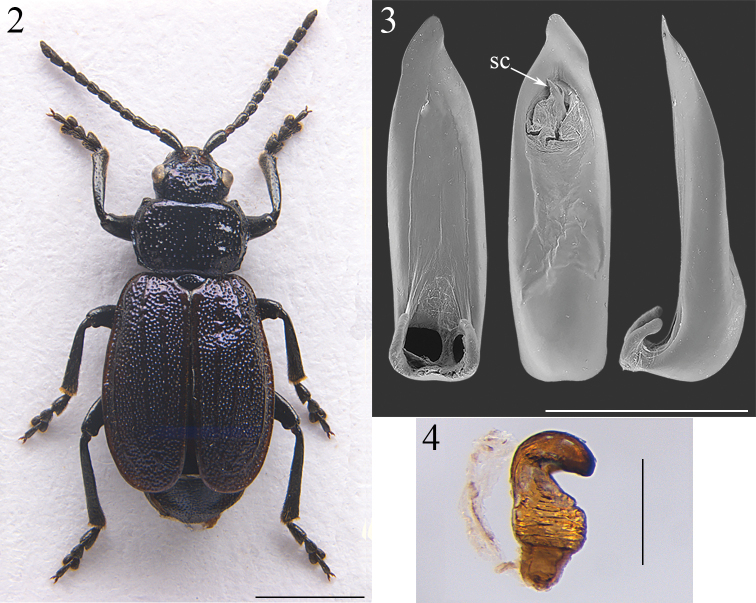
*Belarima violacea* (Lucas) **2** habitus (Tuscan coast, Rosignano Solvay, male) **3** median lobe of aedeagus, from left to right in ventral, dorsal and lateral view (Tuscan coast, Rosignano Solvay) **4** spermatheca (Tuscan coast, Rosignano Solvay). sc: sclerite of the internal sac. Scale bars: 2 mm (**2**); 1 mm (**3**); 0.2 mm (**4**).

The collected specimens show shape, sculpture and color typical of the species, as described by Lucas (1847, 1849). The head, pronotum, scutellum and elytra are clearly metallic violaceous in the male (Fig. 2), while they are green-blue in the female. Both the male and the female are apterous. The apices of the elytra are regularly rounded (Fig. 2), differently from *B. obliqua* where the elytra are more strongly curved along the inner margin than along the outer one (Beenen 2019). The median lobe of the aedeagus (Fig. 3) has a little-sclerotized ventral surface, curved sides, and an asymmetrical apex in ventral view; the apex is regularly constricted and ends in a sharp triangle, differently from *B. obliqua* where it is expanded towards the apex and ends in a blunt triangle (Beenen 2019); the median lobe is straight up to the apex in lateral view; the basal part is swollen dorsally and with lateral hook-shaped extensions ventrally; the sclerite of the internal sac ends in three sharp teeth (Fig. 3). The spermatheca (Fig. 4) has hook-like, thickset cornu not inserted into the nodulus; a globose and wrinkled nodulus, as large as the cornu; and ductus with a very robust and conical proximal part.

#### Biometry.

♂: LB = 7.07 mm; LP = 1.30 mm; WP = 2.21 mm; LE = 4.13 mm; WE = 3.12 mm; LAN = 3.43 mm; LA = 55:20:34:25:25:26:26:30:31:30:41 (right antenna); LAED = 1.9 mm; LE/LP = 3.18; WE/WP = 1.41; WP/LP = 1.70; WE/LE = 0.75; LAN/LB = 0.48; LE/LAED = 2.17. ♀: LB = 6.80 mm; LP = 1.32 mm; WP = 2.24 mm; LE = 4.06 mm; WE = 3.09 mm; LAN = 3.44 mm; LA = 56:23:35:27:24:31:23:25:30:30:40 (right antenna); LSP = 0.32 mm; LE/LP = 3.08; WE/WP = 1.38; WP/LP = 1.70; WE/LE = 0.76; LAN/LB = 0.50; LE/LSP = 12.69.

#### Distribution.

Algeria: Lac Tonga, surroundings of Lacalle [= El Kala]; Djurdjura; Annaba [= Bône] (Lucas 1849; Joannis 1865; Warchalowski 2010); Morocco (Jolivet 1967, indefinite locality), and Tunisia: Aïn Draham and Téboursouk (Beenen 2019); Italy: Tuscany (Livorno), Rosignano Solvay (Fig. 1).

#### Ecological data.

The only data available on the host plants of *B. violacea* are by Jolivet (1967, as *Galeruca violacea*): *Pulicaria odora* L. (Asteraceae), *Rumex acetosella angiocarpus* Murb. and *Rumex scutatus induratus* Boissier (Polygonaceae). However, these data require future confirmation.

## Discussion

The occurrence of this North African species on the Tuscan coast is difficult to interpret. The possible hypotheses to explain these findings are essentially three:

- relict population of a wider past distribution in the north-western Mediterranean. This hypothesis is rather unlikely, considering that other populations, in this case, would have had to survive in suitable areas of the Mediterranean. However, despite the intense research activity that has always involved this area, no other sites of occurrence of the species are known, excluding the North African ones;
- occurrence due to passive anthropogenic transport between North Africa and this Tuscan locality. This hypothesis cannot be ruled out, although unlikely. The only sites close to the collecting locality that could constitute entry points for a possible passive anthropogenic introduction are the commercial port of Vada (distance 2.4 km S) and the tourist port of Cala de’ Medici (distance 1.6 km N);
- possible colonization of the Tyrrhenian and Ligurian coasts through recent, or relatively recent, passive diffusion of this species from North Africa, vehiculated by assemblages of vegetal debris transported by the sea, possibly along the northern flow branch of the Algerian current. This marine current flows anticlockwise around the Tyrrhenian Sea along the coasts of Sicily and the Italian Peninsula before entering the Channel of Corsica (El-Geziry and Bryden 2010) (Fig. 1). Similar distributions due to possible vehiculation by Mediterranean marine currents have also been hypothesized for other species of Coleoptera (cf. Audisio et al. 2011).

Any hypothesis of active displacement can be excluded considering that the species is unable to fly. Future collecting in this Tuscan locality may provide information on the stability, or otherwise, of populations of *B. violacea* on the Italian coasts. In addition, new material would allow molecular analysis of the specimens and comparison with specimens from the North African populations, to evaluate their genetic distances.

## Supplementary Material

XML Treatment for Belarima violacea
